# Neuroimaging-derived brain age is associated with life satisfaction in cognitively unimpaired elderly: A community-based study

**DOI:** 10.1038/s41398-022-01793-5

**Published:** 2022-01-20

**Authors:** Daichi Sone, Iman Beheshti, Shunichiro Shinagawa, Hidehito Niimura, Nobuyuki Kobayashi, Hisashi Kida, Ryo Shikimoto, Yoshihiro Noda, Shinichiro Nakajima, Shogyoku Bun, Masaru Mimura, Masahiro Shigeta

**Affiliations:** 1 Department of Psychiatry, Jikei University School of Medicine, Tokyo, Japan; 2 Department of Human Anatomy and Cell Science, University of Manitoba, Winnipeg, Canada; 3 Department of Neuropsychiatry, Keio University School of Medicine, Tokyo, Japan; 4 Department of Virology, Jikei University School of Medicine, Tokyo, Japan

**Keywords:** Neuroscience, Biomarkers

## Abstract

With the widespread increase in elderly populations, the quality of life and mental health in old age are issues of great interest. The human brain changes with age, and the brain aging process is biologically complex and varies widely among individuals. In this cross-sectional study, to clarify the effects of mental health, as well as common metabolic factors (e.g., diabetes) on healthy brain aging in late life, we analyzed structural brain MRI findings to examine the relationship between predicted brain age and life satisfaction, depressive symptoms, resilience, and lifestyle-related factors in elderly community-living individuals with unimpaired cognitive function. We extracted data from a community-based cohort study in Arakawa Ward, Tokyo. T1-weighted images of 773 elderly participants aged ≥65 years were analyzed, and the predicted brain age of each subject was calculated by machine learning from anatomically standardized gray-matter images. Specifically, we examined the relationships between the brain-predicted age difference (Brain-PAD: real age subtracted from predicted age) and life satisfaction, depressive symptoms, resilience, alcohol consumption, smoking, diabetes, hypertension, and dyslipidemia. Brain-PAD showed significant negative correlations with life satisfaction (Spearman’s rs= −0.102, *p* = 0.005) and resilience (rs= −0.105, *p* = 0.004). In a multiple regression analysis, life satisfaction (*p* = 0.038), alcohol use (*p* = 0.040), and diabetes (*p* = 0.002) were independently correlated with Brain-PAD. Thus, in the cognitively unimpaired elderly, higher life satisfaction was associated with a ‘younger’ brain, whereas diabetes and alcohol use had negative impacts on life satisfaction. Subjective life satisfaction, as well as the prevention of diabetes and alcohol use, may protect the brain from accelerated aging.

## Introduction

The human brain changes with age, and aging is known to be associated with alterations of brain function and sometimes neurodegenerative diseases. The aging process of the brain is biologically complex and varies widely among individuals, and such variability in brain age may contribute to the diversity of individual minds and neuropsychiatric disorders. In recent years, the advances in machine learning and its applications have been remarkable, and it is currently possible to predict an individual’s brain age using structural and/or functional brain images [1, 2]. A neuroimaging-derived brain-age prediction model learns the patterns of image data of many healthy people and their actual ages, and when new image data are input into the system, it can predict a given brain’s age based on the learning model. Such a model can generally show an accuracy of approx. 5 years for adults and <1 year for children and adolescents [1]. The application of brain-age prediction models has been spreading rapidly in recent years to explore the relationship between brain aging and neuropsychiatric disorders, including psychosis, dementia, and epilepsy [3–7].

There have also been several applications of brain-age prediction to general populations for the estimations of individual health and related factors of brain aging, in which some risks for accelerated aging (e.g., diabetes) and beneficial factors (e.g., composing music, meditation) have been suggested [8–12]. In light of the growing populations of the elderly in many parts of the world, the well-being of humans in the so-called ‘golden years’ of life is an issue of great interest. Mental health may play a key role in ‘positive aging,’ [13] and a brain-age prediction model may also become a useful surrogate marker reflecting healthy aging in older individuals.

A large cohort of the elderly in the UK reported that the estimated ages of the participants’ brains were associated with fluid intelligence and allostatic load, as well as mortality [12]. In terms of brain aging, mental states and various lifestyle-related diseases may thus interact each other in complex ways, and their relationship in late life would similarly be biologically complex. To establish the usefulness of brain-age prediction as a surrogate biomarker of mental health in late life, further investigations using various community-based cohorts and populations of different ethnicities may be informative. Resilience and depression in particular have been suggested to be positively and negatively associated, respectively with successful aging, which may be confirmed by neuroimaging-based brain-age analyses. In addition, life satisfaction or well-being may also have effects on an individual’s positive aging [13].

Considering the positive effect of mental well-being on healthy aging, we hypothesized that life satisfaction and/or other mental factors may affect the brain’s aging process independently beyond the common lifestyle-related metabolic factors, and that the clarification of such relationships could provide key insights for better health in the elderly. In this cross-sectional observational study, we investigated the relationships between brain aging and relevant mental factors as well as lifestyle-related metabolic diseases in a cognitively unimpaired population of older participants living in their community in Tokyo. We focused on life satisfaction, resilience, and depression and how these factors may be associated with the quality of life in individuals’ later years.

## Materials and methods

### Participants

Data for this cross-sectional study were extracted from a community-based cohort study in Arakawa Ward, Tokyo, namely, the Arakawa 65+ Study, which was also a participant in a multicenter national survey, i.e., the Japan Prospective Studies Collaboration for Aging and Dementia (JPSC-AD) [14]. In brief, the survey was conducted in Arakawa Ward, Tokyo, among 5,800 randomly selected residents out of the ward’s approx. 42,990 local people aged 65–84 as of October 1, 2016, which was approved by the ethical committee of Keio University School of Medicine. Of those, 1458 participants agreed to the survey with written informed consent, and a final total of 1054 individuals completed a lifestyle and health questionnaire, a face-to-face interview, and magnetic resonance imaging (MRI) scans. The present study’s inclusion criteria were thus (i) residents of Arakawa Ward aged 65–84 years who (ii) agreed to participate in the Arakawa 65+ Study and completed the questionnaire and face-to-face interview and underwent MRI scans. The demographical and clinical assessments were performed between January 2017 and March 2018, and the mean ± SD interval between the clinical assessment and MRI scan was 19.0 ± 13.6 days. The details of this process are described elsewhere [15].

**Fig. 1:**
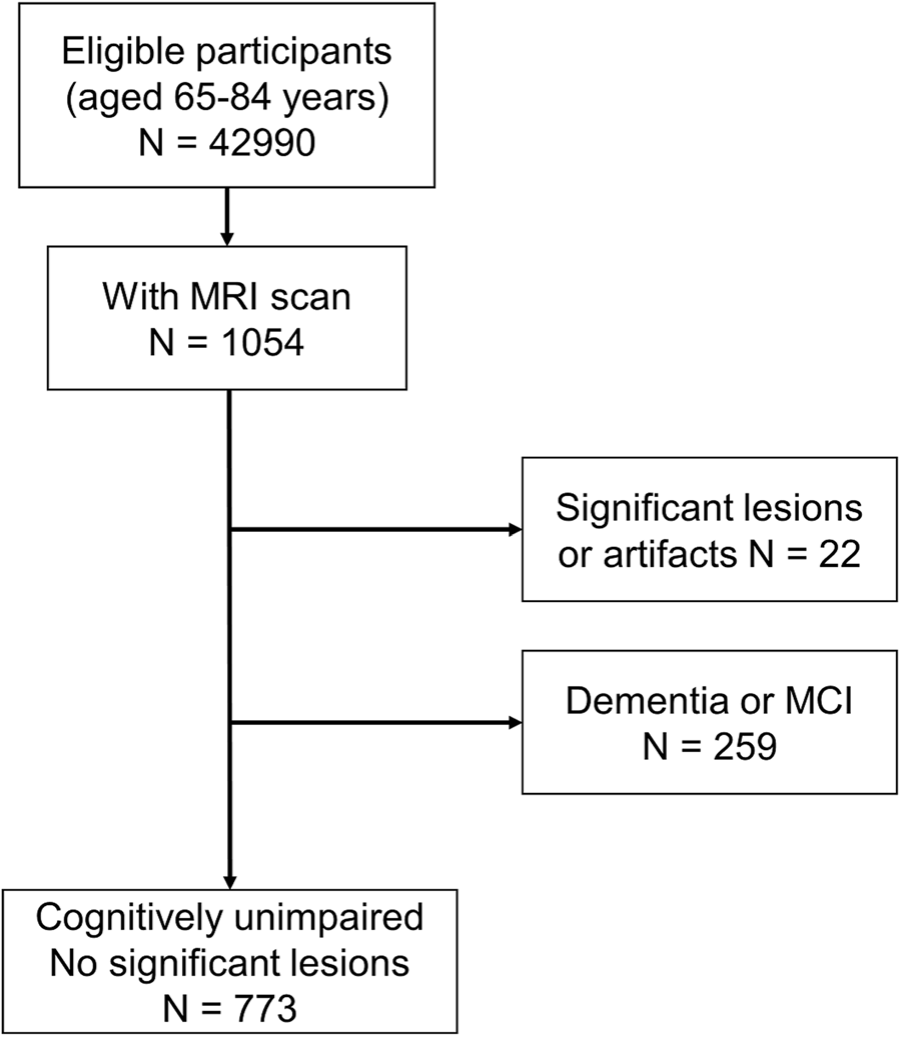
The inclusion process of subjects. A final total of 773 cognitively unimpaired elderly with no visible brain lesions were included.

After a quality check of the MRI findings, we removed 22 participants because of significant artifacts or lesions such as those due to strokes, traumatic contusions, or tumors, which may be problematic for brain age analysis. We excluded another 259 participants with dementia or mild cognitive impairment (Fig. 1), since it is known that cognitive impairment also affects brain aging [3, 16]. The diagnosis of dementia or mild cognitive impairment was based on a comprehensive review of the subject’s cognitive assessment and a face-to-face interview with a psychiatrist or neurologist. Our present investigation’s exclusion criteria were thus as follows: (i) significant structural lesions or artifact that may affect a brain age analysis on visual MRI assessment, and (ii) the presence of dementia or mild cognitive impairment revealed in the comprehensive assessment by a clinician.

A final total of 773 people aged 65–84 years with unimpaired cognitive function and no significant lesions visible on MRI were included in the present analyses.

### MRI acquisition and preprocessing

Brain MRI scans for the participants were performed using a 3-Tesla MRI scanner (Magnetom Spectra; Siemens, Erlangen, Germany) with a 16-channel head–neck coil [15]. A T1-weighted 3D sagittal magnetization-prepared rapid gradient echo sequence was performed with the following parameters: repetition time/echo time, 2300 ms/3.01 ms; flip angle, 9°; voxel size, 1.1 × 1.1 × 1.2 mm^3^; 176 slices; matrix, 256 × 256; field of view, 27 × 27 cm.

The structural brain MRI was processed with the Statistical Parametric Mapping 12 (SPM12: http://www.fil.ion.ucl.ac.uk/spm/software/spm12/) and the Computational Anatomy Toolbox (CAT12: http://www.neuro.uni-jena.de/cat/) running on Matlab2018b (MathWorks, Natick, MA, USA). All 3D T1-weighted MRI scans were normalized using affine followed by non-linear registration, corrected for bias field in homogeneities, and then segmented into gray matter, white matter, and cerebrospinal fluid (CSF) components. We used the diffeomorphic anatomic registration through exponentiated lie algebra (DARTEL) algorithm to normalize the segmented scans into a standard MNI (Montreal Neurological Institute) space. The spatially normalized gray-matter images were then smoothed with a 4 mm full width at half maximum Gaussian kernel and resampled into 8 mm isotropic spatial resolution [7, 17].

### Brain-age prediction

The support vector regression algorithm implemented in MATLAB (i.e., “fitrsvm” function, kernel: linear) was used for predicting the brain age values [6]. We applied the brain age prediction model on the full sample using ten-fold cross-validation. To avoid dimensionality, we used the principal component analysis (PCA) technique within the ten-fold cross-validation. We set the number of principal components at 200. Finally, each subject’s brain-predicted age difference (Brain-PAD: predicted age—chronological age) was calculated.

### Assessment of mental states and lifestyles

The participants underwent the Mini-Mental State Examination (MMSE), the Satisfaction With Life Scale (SWLS) [18], the Resilience Scale [19], and the Geriatric Depression Scale (GDS) [20], which were used to assess cognitive function, life satisfaction, resilience, and depressive symptoms, respectively. The following lifestyle factors and related diseases were described by participants in their responses to the questionnaire: current use of alcohol, current smoking, diagnosis of diabetes, hypertension, and dyslipidemia. As these items may affect brain aging as risk factors [8], we included them in the analysis.

### Statistical analyses

The statistical analyses were performed by SPSS software ver. 25.0. Parametric or non-parametric distributions of variables were examined by the Kolmogorov–Smirnov test. We used Spearman’s rank correlation to analyze the bivariate correlations between the participants’ Brain-PAD and their mental/psychological scores.

We also performed a multiple regression analysis to identify factors that were independently associated with increased or decreased brain aging. The Brain-PAD was set as the dependent variable, and the independent predictor variables included the MMSE score, the SWLS score, the Resilience score, the GDS score, current use of alcohol, current smoking, diabetes, hypertension, and dyslipidemia. Chronological age, sex, education level, and total intracranial volume (TIV) [21] calculated by CAT12 were also added in the model to adjust the effect of these variables. The normal distribution of the residuals of the regression model was confirmed by Kolmogorov–Smirnov test. A *p*-value <0.05 was deemed significant.

## Results

### Demographics

The details of the participants’ demographics are provided in Table 1. In total, 320 men and 453 women were included for the analysis, and most of participants achieved an almost perfect score on the MMSE. Alcohol use, smoking, diabetes, and hypertension were present more frequently in the males, and dyslipidemia was more prevalent in the females.

**Table 1:** Demographic data of the participants from the Arakawa 65+ Study, Tokyo, Japan (*n* = 773, 2016).

		Men	Women	*p*
Subjects	*n* (%)	320 (41.4%)	453 (58.6%)	
Age, yrs	Median (IQR)	70.8 (6.9)	72.3 (7.4)	0.034
MMSE^a^	Median (IQR)	29 (3)	28 (3)	0.143
SWLS^a^	Median (IQR)	22 (6.75)	22 (7)	0.341
Resilience^a^	Median (IQR)	123 (25.75)	123 (24)	0.810
GDS^a^	Median (IQR)	3 (4)	2.5 (4)	0.415
Education Level				<0.001
Primary school	*n* (%)	1 (0.3%)	2 (0.4%)	
Secondary school	*n* (%)	41 (12.8%)	51 (11.3%)	
High school	*n* (%)	132 (41.3%)	263 (58.1%)	
University or higher	*n* (%)	146 (45.6%)	137 (30.2%)	
Current use of alcohol^b^	*n* (%)	233 (72.8%)	161 (35.9%)	<0.001
Current smoking^a^	*n* (%)	57 (21.7%)	26 (5.8%)	<0.001
Diabetes^c^	*n* (%)	60 (19.0%)	51 (11.3%)	0.003
Hypertension^d^	*n* (%)	172 (53.9%)	199 (44.1%)	0.007
Dyslipidemia^e^	*n* (%)	109 (34.9%)	208 (46.4%)	0.002

Differences between the men and women were analyzed by Mann–Whiteny’s U-test for continuous variables and the *χ*^2^ test for categorical variables.

*GDS* Geriatric Depression Scale, *MMSE* Mini-Mental State Examination, *SWLS* Satisfaction with Life Scale.

Missing in ^a^1 subject, ^b^4 subjects, ^c^6 subjects, ^d^3 subjects, ^e^13 subjects.

### Brain-age prediction model

**Fig. 2:**
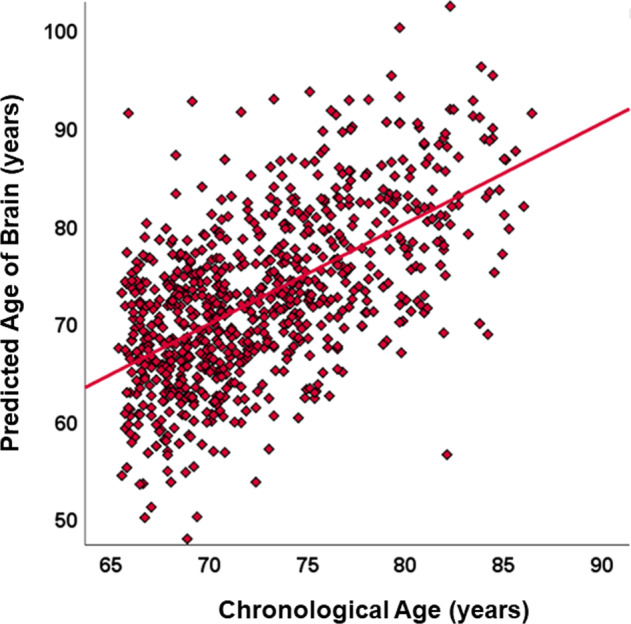
The scatter plot of chronological age and predicted age of brain. The relationship between chronological age and predicted brain age based on structural MRI.

Figure 2 is the scatterplot of the individual participants’ chronological and estimated ages. Our brain-age prediction model showed a mean absolute error of 5.49 years, and the rank correlation coefficient was 0.57 (*p* < 0.001).

### Bivariate correlations with Brain-PAD

**Fig. 3:**
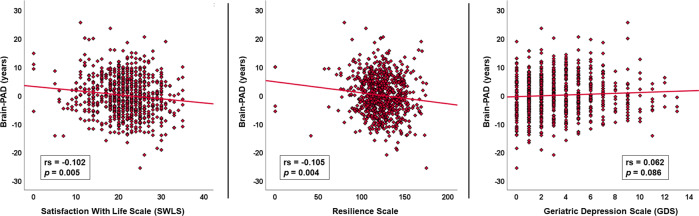
The scatter plots of Brain-PAD and clinical variables. Bivariate correlations of Brain-PAD with life satisfaction, resilience, and depression.

As shown in Fig. 3, decreased Brain-PAD was significantly correlated with both life satisfaction (rs = −0.102, *p* = 0.005) and resilience (rs = −0.105, *p* = 0.004). Depressive symptoms showed an insignificant association with increased Brain-PAD (rs = 0.062, *p* = 0.086).

### Multiple regression analysis

A total of 753 participants with no missing variables were included in the multiple regression analysis. The results are summarized in Table 2. Among the predictor variables, the SWLS score, current use of alcohol, and diabetes were independently associated with Brain-PAD. These factors were independently significant with correction for the TIV, although the TIV was also related (*p* = 0.029). The resilience score also showed a trend-level insignificant association (*p* = 0.098). According to the multiple regression analysis results, the existence of diabetes increases the brain age by 2.253 year and the existence of alcohol use increased the brain age by 1.098 years, while the brain age may decrease by 0.114 years with each single point of the SWLS score. Based on the standardized beta value, which represents the relative effects among the variables, these three factors showed similar effect sizes, i.e., around 0.08–0.11.

**Table 2:** Results of multiple regression analysis with the Brain-PAD score as a dependent variable.

Variable	Unstandardized beta	SE	95%CI	Standardized beta	*t*-value	*p*-value
Age	0.047	0.052	−0.057 to 0.151	0.034	0.908	0.364
Sex^a^	0.272	0.708	−1.144 to 1.689	0.019	0.384	0.701
Education^b^	0.077	0.388	−0.699 to 0.853	0.007	0.198	0.843
MMSE	0.073	0.135	−0.196 to 0.342	0.02	0.541	0.589
**SWLS**	**−0.114**	**0.055**	**−0.223 to −0.005**	**−0.093**	**−2.083**	**0.038**
Resilience	−0.023	0.014	−0.051 to 0.005	−0.067	−1.657	0.098
GDS	−0.027	0.111	−0.249 to 0.195	−0.01	−0.243	0.808
**Alcohol**^c^	**1.098**	**0.535**	**0.028 to 2.167**	**0.08**	**2.052**	**0.040**
Smoking^c^	1.276	0.822	−0.369 to 2.920	0.058	1.552	0.121
**Diabetes**^c^	**2.253**	**0.726**	**0.802 to 3.705**	**0.113**	**3.104**	**0.002**
Hypertension^c^	0.624	0.506	−0.387 to 1.635	0.045	1.235	0.217
Dyslipidemia^c^	0.001	0.511	−1.021 to 1.023	0	0.002	0.998

Bold values indicate statistical significance.

*MMSE* Mini-Mental State Examination, *SWLS* Satisfaction With Life Scale, *GDS* Geriatric Depression Scale.

^a^Categorized males as ‘0’ and females as ‘1’.

^b^Categorized from ‘1’ to ‘4’ according to the classification in Table 1.

^c^Categorized absence as ‘0’ and presence as ‘1.’

Regarding the possibility of multicollinearity, all of the absolute values of bivariate correlation coefficients were at most around 0.5 (e.g., −0.517 between the SWLS and GDS scores, and 0.446 between the SWLS and Resilience scores).

## Discussion

We explored relevant factors for brain aging in late life and observed the SWLS score, the use of alcohol, and diabetes are independent predictors. Is particularly notable that life satisfaction is related with younger brain age independently of alcohol use, metabolic diseases, depression, and other psychosocial factors. Our findings may provide community-level evidence about ways to keep our brains younger in our later years.

For a comparison with our present findings, we searched the Medline database for similar research, particularly regarding brain aging in general older populations and relevant factors for positive aging. Life satisfaction or well-being is a crucial issue particularly in older people’s lives, which are known to be improved by psychosocial or technology-based interventions [22, 23]. Social activities and computer-based training, in particular, were suggested to be effective [22, 23]. As our present analyses identified life satisfaction as an independent predictor for younger-appearing brains, promoting such social interventions may well contribute to not only well-being but also anti-aging of the brain, which would be consistent with the negative effect of worrying and rumination on brain aging reported by a recent study [24]. It is also suggested that life satisfaction is closely associated with wisdom [25]. A positive effect of fluid intelligence on brain age was described [12]; thus, wisdom and life satisfaction may interact with each other and make our brains younger (although we did not estimate the participants’ intelligence in this study).

We also identified resilience as an important factor for a younger brain, though it was not independently significant. A questionnaire study noted that resilience was an important trait for successful aging [26], and our study result provided further evidence regarding the role of resilience in late life. Considering the nature of resilience and life satisfaction, resilience may serve as a potentially effective trait for positive aging, while life satisfaction would reflect subjective feelings about the consequences of people’s current and past lives.

It was suggested that geriatric depression was related to older-appearing brains [16], whereas we found only a trend-level correlation in the bivariate analysis. As reported, depression can be another important factor acting against positive aging [26], and late-life depression is known to worsen cognitive function in the elderly [27–30]. Depression is not only a strong risk factor for dementia [30] but also has the effect of actually decreasing apparent cognitive function [31]. To remove the potential effect of cognitive impairment on brain aging, we enrolled only cognitively unimpaired participants in the present study, and this might have underestimated the effect of depression.

Several other studies have investigated life-associated factors for brain aging [8–11]. Music composition and meditation practice were reported to have help make our brains younger [10, 11]. These activities also positively affect our mental states, which supports the significance of life satisfaction identified in the present study. It is known that engaging various leisure activities can prevent cognitive impairment in the elderly [32], and similar protective mechanisms may underlie brain aging. Our analyses also revealed diabetes and alcohol use as significantly independent predictors for brain aging; both of these factors were also reported in an earlier investigation [8]. Diabetes in particular was strongly associated with increased brain age (Table 2). These lifestyle factors or risk factors of metabolic diseases can often be partially prevented or appropriately treated by medications, and it should thus be possible for us to prevent our brains from accelerated aging. However, even individuals who already have diabetes can improve their brains, since we observed that subjective life satisfaction was significantly associated with brain age independently of diabetes and other factors. Our present findings thus provide evidence suggesting a potential approach to keep our brains young in late life.

This study has several limitations. First, cross-sectional data do not reveal causal relationships, and our perspective on the prevention of accelerated aging is still speculative. A question remains: Does happiness make us healthier, or because we are healthy, we are happier? In addition to the causality, the preventability of the detected risk factors is also controversial. In particular, it is not easy to prevent diabetes in individuals with increased genetic susceptibility.

A second study limitation is that there might be other unknown or unevaluated confounders that affect brain aging in the elderly, although we analyzed a medium sample size from a community-based cohort. Our survey did not include common diseases other than those described (e.g., as chronic inflammatory diseases and kidney diseases) or drugs for these conditions, which may affect the brain aging process. Interestingly, a recent study reported increased body mass index (BMI) as a risk factor for accelerated brain aging in a young European cohort with first-episode psychosis [33]. Compared to western countries, the prevalence of overweight and obesity in older people is much lower in Japan [34], however. In nature, a lower BMI (e.g., <18) is not always better for health, and this would be particularly applicable to older Japanese people. We thus considered BMI unsuitable for the linear statistical analysis in this study. In addition, even though we excluded participants with cognitive impairment, it can be expected to be difficult to completely remove the potential existence of early preclinical neurodegenerative pathology, which may lead to reverse causality.

Third, some of the present lifestyle data (such as alcohol use and smoking) were not detailed but only binary. In addition, as we only included cognitively unimpaired people, our findings may not be applicable to elderly individuals with dementia or mild cognitive impairment. There is a strong relationship between aging and neurodegenerative dementia [35], and accelerated brain aging was suggested as an important risk factor for cognitive decline [2, 3]. The prevention of accelerated aging is thus a relevant topic for brain health, and we speculate that it might be possible for us to prevent dementia or cognitive impairment by keeping our brains younger. In this regard, our study may provide significant insights, despite its limitations.

Finally, despite the statistical significance, the effect size of each variable was not large. As we described, the biological process of aging is complex and may vary among individuals. Further investigations using longitudinal and larger cohorts with more comprehensive data may resolve these limitations more clearly.

In conclusion, our analyses identified life satisfaction, diabetes, and use of alcohol as significantly independent predictors for brain age in a community-based elderly cohort. Resilience may also be important. It is possible that people could keep their brains younger by improving their subjective life satisfaction, avoiding alcohol use disorder, and preventing the development of diabetes.
